# Selective Cooperation in Early Childhood – How to Choose Models and Partners

**DOI:** 10.1371/journal.pone.0160881

**Published:** 2016-08-09

**Authors:** Jonas Hermes, Tanya Behne, Kristin Studte, Anna-Maria Zeyen, Maria Gräfenhain, Hannes Rakoczy

**Affiliations:** 1 Institute of Psychology, University of Göttingen, Waldweg 26, D-37073 Göttingen, Germany; 2 Leibniz ScienceCampus Primate Cognition, Kellnerweg 4, D-37077 Göttingen, Germany; 3 Department of Child and Adolescent Psychiatry, Psychotherapy and Psychosomatics, University of Leipzig, Liebigstr. 20a, D-04103 Leipzig, Germany; Centre for Coevolution of Biology & Culture, University of Durham, UNITED KINGDOM

## Abstract

Cooperation is essential for human society, and children engage in cooperation from early on. It is unclear, however, how children select their partners for cooperation. We know that children choose selectively whom to learn from (e.g. preferring reliable over unreliable models) on a rational basis. The present study investigated whether children (and adults) also choose their cooperative partners selectively and what model characteristics they regard as important for cooperative partners and for informants about novel words. Three- and four-year-old children (N = 64) and adults (N = 14) saw contrasting pairs of models differing either in physical strength or in accuracy (in labeling known objects). Participants then performed different tasks (cooperative problem solving and word learning) requiring the choice of a partner or informant. Both children and adults chose their cooperative partners selectively. Moreover they showed the same pattern of selective model choice, regarding a wide range of model characteristics as important for cooperation (preferring both the strong and the accurate model for a strength-requiring cooperation tasks), but only prior knowledge as important for word learning (preferring the knowledgeable but not the strong model for word learning tasks). Young children’s selective model choice thus reveals an early rational competence: They infer characteristics from past behavior and flexibly consider what characteristics are relevant for certain tasks.

## Introduction

Cooperation is an essential social-cognitive skill that constitutes human society, is uniquely human on its sophisticated levels and emerges in early childhood [1,2]. Young children already engage in cooperative activities with adults and peers [3–5] and understand essential aspects of joint collaboration: By 2 years, children engage in collaborative activities regardless of whether or not a partner is needed to achieve a physical goal and are sensitive to whether or not a partner intends to cooperate [6]. And from 3 years, children regard joint commitments of collaborative engagements as binding [7] and coordinate two complementary roles in cooperative problem-solving [8]. However, successful cooperation not only depends on children fulfilling the commitments they entered into with their partners, but also on the appropriate choice of individuals to cooperate with. Although little is known about how children choose cooperative partners, indirect evidence from previous research suggests that children are sensitive to a cooperative partner’s intentions, as they are more reluctant to re-initiate a cooperative activity with a previously unwilling partner than with a previously unable partner [6] and engage in direct and indirect reciprocity by sharing selectively with those who had shared themselves [9]. And chimpanzees, too, choose cooperative partners that have proven competent in the past [10]. This raises the question of how children (or apes) make such choices. Do they evaluate relevant characteristics of potential partners and selectively choose who to collaborate with on that basis?

What we do know, however, is that children are quite selective when it comes to the question whom to learn from. Extensive recent work on children’s selective social learning has shown that preschoolers do not just blindly pick up information from anyone indiscriminately, but selectively request and endorse information from partners with certain characteristics (see [11–14] for reviews). When learning the labels of novel objects, for example, children from age 3–4 prefer to learn from knowledgeable over ignorant, confident over unconfident, previously reliable over unreliable and adult over peer models [15–17].

But it remains unclear on what social cognitive underpinnings this selectivity rests. Is it a rational process, similar as it would be required for the reasonable choice of partners for a cooperative activity? Do children systematically generalize from perceived model characteristics (e.g. previous linguistic accuracy) to the expected usefulness of the model for specific future problems (e.g. finding out names of novel objects)–much like adults would often reason (“She was consistently accurate, so she seems reliable, so I should trust her when needing novel information”)? Or is it a more heuristic and less systematic process, perhaps having to do with more global impression formation in the style of halo-effects? Rather than perceiving some model as reliable, or competent in some specific way, children might form very global impressions of someone as simply positive across the board, leading to some very unspecific preference for that person regardless of the problem at hand. So someone previously shown to be nice, say, or a good singer, might be perceived as globally positive and therefore preferred for any task. A third possibility, that young children simply extrapolate future behavior based on the past track record of that specific behavior seems implausible in the light of many studies that have found some transfer from one type of behavior shown by a model to predictions about or evaluations of another kind of behavior by that model [18–21].

The question of whether selective learning—as documented abundantly in 4-year-olds—is based on rational inductive inferences or rather on global, heuristic impression formation is still unresolved. But divergent findings suggest that preschoolers use both strategies in certain situations: On the one hand there are results that seem to support global, halo-style impression formation. For example, in some studies children expected models that previously were more accurate to be nicer in future too [18], and previously stronger models to be more accurate [22]. On the other hand, however, several studies have documented that children generalize competences in a rational way. When, for example, confronted with a strong and a knowledgeable model, preschoolers rationally choose between the two for knowledge-relevant or strength-relevant problems [23]. Similarly, they differentiate between domains of knowledge: when confronted with experts from different domains (e.g. toy labeler vs. toy fixer or doctor vs. car mechanic), they direct questions reasonably to the according expert [24,25]. Preschoolers have even shown to be flexible and context-sensitive in their model recruitments: while 4-year-olds usually prefer adults over peers as a source for learning new words, such preferences can be reversed when an adult model has previously proved unreliable [17]. Moreover, children trust adults over children for food questions (where adults usually know more) but trust children over adults for toy questions (where children usually know more; see [26]).

In the present study, we intend to explore the cognitive underpinning of children’s selective recruitment of social partners for cooperative activities using the paradigms established to examine children’s epistemic trust.

The results from studies on epistemic trust indicate that children—at least under certain circumstances—choose models rationally. But obviously in this research, very clearly circumscribed tasks (such as learning words for novel objects) were used, that were quite clearly connected to certain characteristics (accuracy, knowledge). The requirements for a cooperative partner are however less clear cut. What would characterize a rational choice of a cooperation partner? Our intuition is that for successful cooperation a much broader range of model characteristics are diagnostic than for simple, circumscribed tasks, such as word learning. Thus for a novel word learning task, for example, a model’s previous accuracy in object labelling is clearly diagnostic and relevant whereas other characteristics such as a model’s relative strength or weakness is not. However, in the case of real life interactions, co-operations in particular, things are much more complex. Say you are moving house and have to move a heavy wardrobe from one place to the next. Obviously, you would preferentially recruit a strong partner over a weak one, but a much broader range of factors might be important, such as affiliation or a potential partner’s knowledge or reliability, and perhaps many more. In contrast to the word learning task, it is not simply a question of requesting or endorsing a piece of information, but of interacting and coordinating with another person. Thus when recruiting partners and choosing models based on their past track record, what kind of inferences and generalizations are justified depends on the scope of model characteristics and on the scope of requirements of the tasks involved.

Against this background, in the present study we tested whether the generalizations children draw from a model characteristic vary as a function of different tasks. We expected that children regard a broad range of characteristics as predictive for the choice of a cooperative partner, whereas for the choice of an informant to learn labels for novel objects from only a narrow range of characteristics, clearly connected to the problem (such as knowledgeability), should be regarded as diagnostic.

To test this in the current study we employed a design similar to Fusaro et al. [22] in manipulating the two characteristics labeling accuracy and strength. To explore the different scopes of inferences that children draw from characteristics such as strength and word knowledge, Fusaro et al. [22] confronted children with a good and a bad lifter (strength condition) or a good and a bad labeler (accuracy condition) and later children chose between those models in a labeling and a strength task and answered several trait and behavioral prediction questions. In the current study we used a similar design to explore children’s assessment of potential cooperative partners. Our focus here is on whether a broader range of characteristics is regarded as predictive for cooperative tasks as compared to clearly circumscribed solitary tasks. In the current study we therefore adopted the general design of Fusaro et al. [22], but instead of their pure strength task, we developed cooperation tasks in which physically taxing activities needed to be done together. For the more clearly circumscribed solitary task we used the established word learning paradigm that has commonly been used in selective trust studies. Labeling accuracy and physical strength were chosen as model characteristics because these do not obviously overlap and can be demonstrated in a way easily comprehensible for young children [22,23].

In the familiarization phase, participants thus saw contrasting pairs of models that differed either in their physical strength (strong/weak) or in their labeling accuracy (accurate/inaccurate). In the test phase children were then confronted with two types of problems: In *word learning tasks*, participants were shown novel, unknown objects and chose from which model to endorse information about them. The *word learning tasks* were thus clearly connected to labeling accuracy, but not to strength. In *cooperation tasks*, participants were confronted with a problem that could only be solved by two people and that required both physical force and skillfulness. The *cooperation tasks* were thus clearly connected to physical strength but not directly to labeling accuracy. If participants regard a broad range of characteristics as relevant for a cooperative partner and, in contrast, regard only clearly connected and circumscribed characteristics as diagnostic for successful word learning, they would prefer both the strong (over the weak) and the accurate (over the inaccurate) model for a cooperative task. In contrast, for the word learning task, they would prefer the accurate over the inaccurate model but should not systematically prefer the strong or the weak model.

First, in a pilot study we tested this intuition of rational selective choice of informants and cooperative partners in adults and thereafter conducted an age-appropriate version for 3- and 4-year-old children. These age groups were specifically chosen because selective learning and trust have often been found to emerge between the ages of 3 and 4 (e.g. [16,21]).

### Ethics Statement

Both the pilot study and the main study were conducted in accordance with the Declaration of Helsinki and the Ethical Principles of the German Psychological Society (DGPs), the Association of German Professional Psychologists (BDP), and the American Psychological Association (APA). They involved no invasive or otherwise ethically problematic techniques and no deception (and therefore, according to national jurisdiction, did not require a separate vote by a local Institutional Review Board; see the regulations on freedom of research in the German Constitution (§ 5 (3)), and the German University Law (§ 22)).

## Pilot Study (Adults)

### Participants

Fourteen adults (*M* = 22 years, age range: 19–29 years, 12 female) participated. They were mostly undergraduate psychology students and were recruited through public announcements.

### Design and procedure

After receiving information about the procedure and the main aim of the study (i.e.to compare adults’ and children’s responses) participants gave oral consent and were then tested individually in a quiet room. The adults were shown the material designed for the child study in an adapted computerized version using pictures and short videos. The sessions lasted approximately 15 min and consisted of a familiarization phase showing the contrast between the two puppets and two test blocks, one with cooperation tasks and one with word learning tasks (with order of blocks systematically varied across participants). Participants wrote down their answers to test questions on a pre-defined answer sheet.

The study followed a 2x2 design. The domain in which models were familiarized (strength or accuracy) was varied between subjects whereas the type of test items (word learning and cooperation) was varied within subject.

#### Familiarization phase

Participants were randomly assigned to one of two familiarization conditions, the *accuracy condition* and the *strength condition*. In the *accuracy condition* participants saw four slides, in each of which two puppet models named a familiar object (e.g. a ball, see S1 Table). One model consistently demonstrated knowledge by naming the objects accurately in speech bubbles whereas the other model consistently demonstrated ignorance by naming the objects incorrectly. In the *strength condition*, participants saw four slides in each of which two puppet models were confronted with a task requiring physical strength (see S2 Table). One puppet demonstrated strength—as indicated by the sentence “Max has no problem in lifting this …” typed beside him. The other puppet demonstrated weakness as indicated by the sentence “Toni does not succeed in lifting this …” typed beside him. After the familiarization phase two comprehension questions were asked. In the accuracy condition we asked who said more/fewer names correctly while in the strength condition, we asked who has more/less strength.

#### Test Blocks

In the word-learning test block (four trials) the participant saw a slide with a picture of an unknown object (see S1 Fig) and was asked if she knew the object. Subsequently both puppets labelled the objects with different novel labels using speech-bubbles and the participant was asked to choose between the two labels.

In the cooperative test block (four trials) the participant saw a short video clip in which a single experimenter introduced and explained the tasks each involving an unknown apparatus (see S3 Table for pictures and details about the information provided). Each apparatus contained hidden toy animals and two persons were required to cooperate in order to retrieve them, with one of the roles requiring some physical force. For example, one apparatus was a wooden box with a cord on the cover plate. The toy animals in the box could be retrieved if one person pulled on the cord (requiring some force), while the other person took out the animals. The participant was asked to choose a puppet to cooperate with. As these materials were designed primarily for child participants in the main study, the adults were explicitly instructed to imagine themselves in the physical (but not cognitive!) position of a small child when thinking about the cooperation problems.

### Results and Discussion

**Fig 1:**
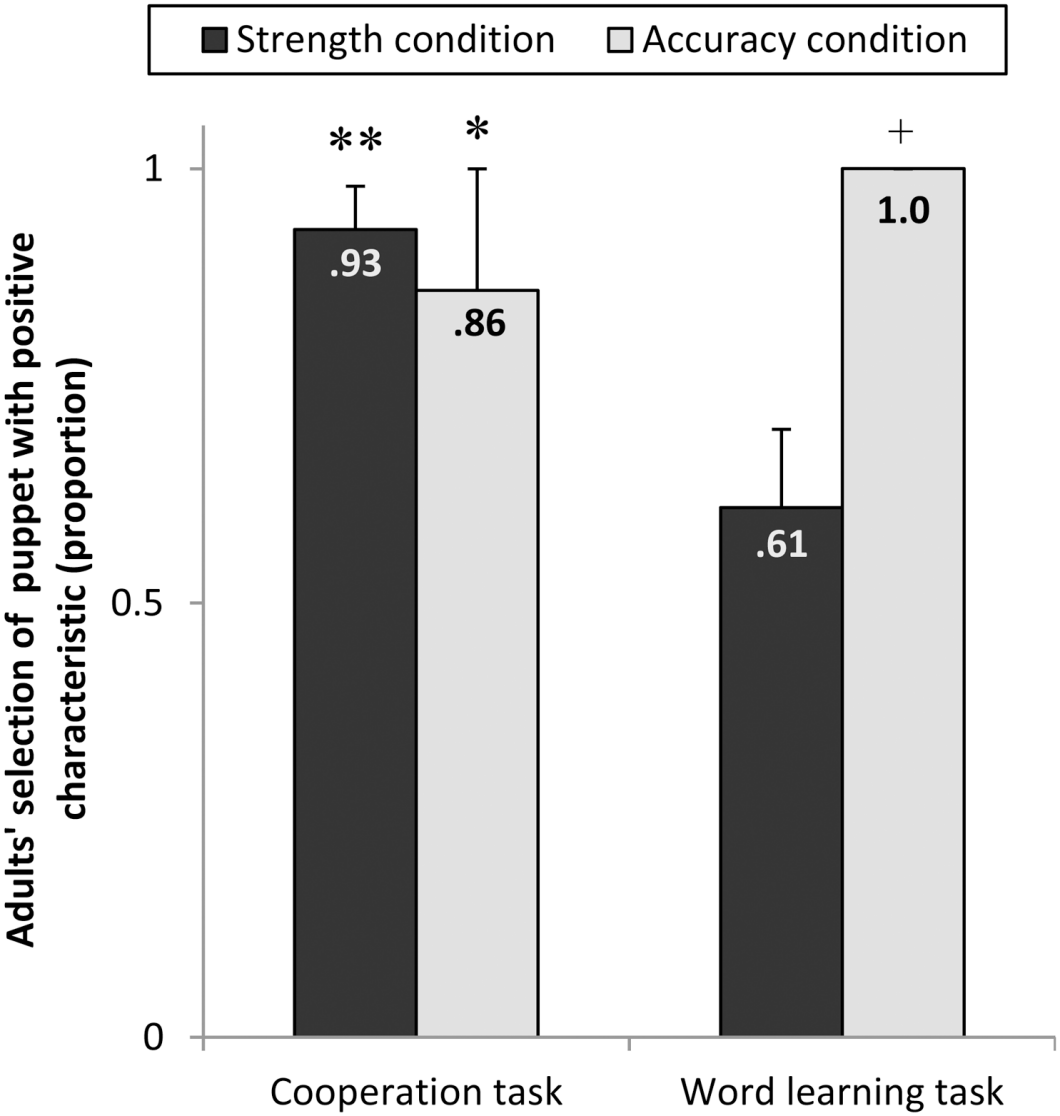
Adults’ selection of the puppet with the positive characteristic. Adults’ selection of the puppet with the positive characteristic as a function of task and familiarization condition. Significantly above-chance choice of the model with the positive characteristic (accurate/strong) is marked by asterisks (*p < .05 and **p < .01, one-sample t-tests). +: no parametrical test was applicable due to a lack of variance. Error bars show standard errors.

The proportions of trials in which adults selected the strong/accurate puppet are illustrated in Fig 1. A mixed ANOVA with type of task as within-subject factor and familiarization condition as between-subject factor on the choice of the puppet with the positive characteristic resulted in an interaction between type of task and familiarization condition (*F*(1,12) = 6.04, *p* < .05, partial η^2^ = .36) and no main effects. Comparisons of the adults’ choice of the puppet with the positive characteristic to chance level revealed that in the word learning tasks they preferred the accurate puppet’s label in every single case, but they did not prefer the strong puppet’s labels above chance (*t*(6) = 1.16, *p* = .29). In contrast, for the cooperation tasks they preferred both the strong puppet (*t*(6) = 9.3, *p* < .01, *d* = 3.5) and the accurate puppet (*t*(6) = 2,5, *p* < .05, *d* = .95) above chance.

Thus, adult participants regarded a broader range of characteristics as relevant for the choice of a cooperative partner, but only clearly circumscribed characteristics as important for word learning. The main study will tell whether 3- and 4-year-olds already show the same pattern of rational selective inferences as adults do.

## Main Study (Children)

### Participants

Thirty-two 3-year-old children (*M* = 44 months, range: 39–47 months, 15 girls) and thirty-two 4-year-old children (*M* = 51 months, range: 48–55 months, 16 girls) were included in the final sample. All children were native German speakers and they were recruited from a database of parents who had volunteered to participate in studies on child development and came from mixed socio-economic backgrounds. Parents gave their written consent for the participation of their children. Fourteen additional children were excluded from analyses due to experimenter error (*n* = 4), failure to answer the comprehension questions after two repetitions (*n* = 5) or uncooperativeness/extreme shyness (*n* = 5).

### Design and Procedure

Children were tested individually in a quiet room, either in their daycare center or in the University child lab. In the lab, children were tested without the accompanying parent being present in the room during the test session. Each session was videotaped. For all children, the session consisted of i) a warm-up ii) a familiarization phase showing the contrast between the two models, and iii) two test blocks: one with cooperative tasks and one with word-learning tasks.

The study followed a 2x2 design. The domain in which models were familiarized (strength or accuracy) was varied between subjects whereas the type of test items (word learning and cooperation) was varied within subject.

#### Warm-up

All testing was done by two experimenters, one who played the two puppets that served as models and one who interacted with the child. Both experimenters played with the child until she felt comfortable and then the two puppets were introduced. We decided to use puppet models (as is typically done in research on selective trust) in order to assure a standardized procedure by restricting differences between the models to the manipulated aspect.

#### Familiarization phase

Children were randomly assigned to one of the two familiarization conditions: The *accuracy condition* and the *strength condition*. In each condition children saw two models. In the manipulation of accuracy we followed a standard procedure commonly used in selective trust studies and we adopted this procedure for the manipulation of strength, using similar methods as in Fusaro et al. [22]. In the *accuracy condition*, children saw the two puppets label the same four familiar objects as in the pilot study (see S1 Table). For each one, the puppets were asked in turn what the object (e.g., a ball) was called. One puppet demonstrated knowledge by consistently naming the objects accurately (e.g. “This is a ball”). The other puppet demonstrated ignorance by consistently naming the same objects inaccurately (e.g. “This is a shoe”).

In the *strength condition* the puppets were confronted with the same four tasks requiring physical strength as in the pilot study (see S2 Table). For example, a heavy suitcase was shown and the puppets were each asked “Can you lift this suitcase?” One puppet consistently demonstrated strength by succeeding in the tasks and saying “That’s easy, I’m good at that”, whereas the other puppet demonstrated weakness by never succeeding and always saying “Ups, that’s tough. I’m not good at that.” The assignment of puppets to positive and negative characteristics was counterbalanced across subjects, and the order in which puppet acted/labeled was counterbalanced across trials for each subject.

#### Comprehension questions

The same two comprehension questions as in the pilot study were asked to assess whether the child understood the performance shown by the puppets. If at least one comprehension question was answered incorrectly, the familiarization phase was extended by repeating one or at most two familiarization trials followed by the same comprehension questions. If these were still not both answered correctly after the repetitions, the child was excluded from analyses. Twelve children needed additional familiarization trials before they provided correct judgments.

#### Test Blocks

Then all children participated in two test blocks, one with word-learning and the other with cooperative tasks. The order of the task blocks was counterbalanced (across children and conditions), as was the order of tasks within each block. Before the second test block, there was a short reminder phase consisting of two further familiarization trials. The reminder trials (for objects see S1 and S2 Tables) followed the same format as in the initial familiarization and were followed by the same comprehension questions. In the reminder phase six children needed one or two additional familiarization trials, before passing the comprehension check. In total, 16 children needed one or more repetitions in the initial familiarization and/or in the reminder familiarization phase. When these 16 children were excluded from analyses, the pattern of results remained the same.

In the word-learning test block (four trials) the child was presented a picture of an unknown object (see S1 Fig) and asked whether she knew what the object was called. If a child felt she was required to answer and guessed a label for the object, this label was doubted by the experimenter saying “I don’t think that’s a (…)”. After the child was asked which puppet she would like to ask for an object label, both puppets provided different novel labels for the object (in counterbalanced order) and the experimenter repeated the labels provided by the puppets. As the crucial test question, the child was then asked to choose between the two labels (e.g. “[Name of puppet A] said that’s a Dreto. [Name of puppet B] said that’s a Taki. What do you think this is, a Dreto or a Taki?”). Over the 4 trials, the proportion of trials in which children endorsed the label of the puppet with the positive characteristic (accurate/strong) was computed as the basis for subsequent statistical analyses.

In the cooperative test block (four trials) the experimenter introduced the same unknown apparatuses as in the pilot study to the child (see S3 Table). On each trial, the experimenter explained the apparatuses and then asked the child with which puppet she would like to cooperate on the apparatus. Over the 4 trials, the proportion of trials in which children chose the puppet with the positive characteristic (accurate/strong) was computed as the basis for subsequent statistical analyses.

### Coding procedure and reliability

All test sessions were coded from videotape by one rater with respect to three types of measures:

- Comprehension questions: Identification of the puppet with the positive and negative characteristic by pointing to and/or naming the puppet (possible scores: 0–2).
- Cooperation task: Number of trials in which the child choses the puppet with the positive characteristic as a partner by pointing to or saying the puppet’s name (possible scores: 0–4).
- Word learning task: Number of trials in which the child uses the label provided by the puppet with the positive characteristic (possible scores: 0–4).

A second rater blind to the hypotheses of the study coded the videos of 12 randomly chosen children. Inter-rater reliability was perfect for the comprehension questions (*κ* = 1.0) and very good concerning children’s scores on the cooperation tasks (*κ* = .88) and on the word learning tasks (*κ* = .77).

The datasets can be found in S1 Dataset (pilot study) and S2 Dataset (main study).

### Results

**Fig 2:**
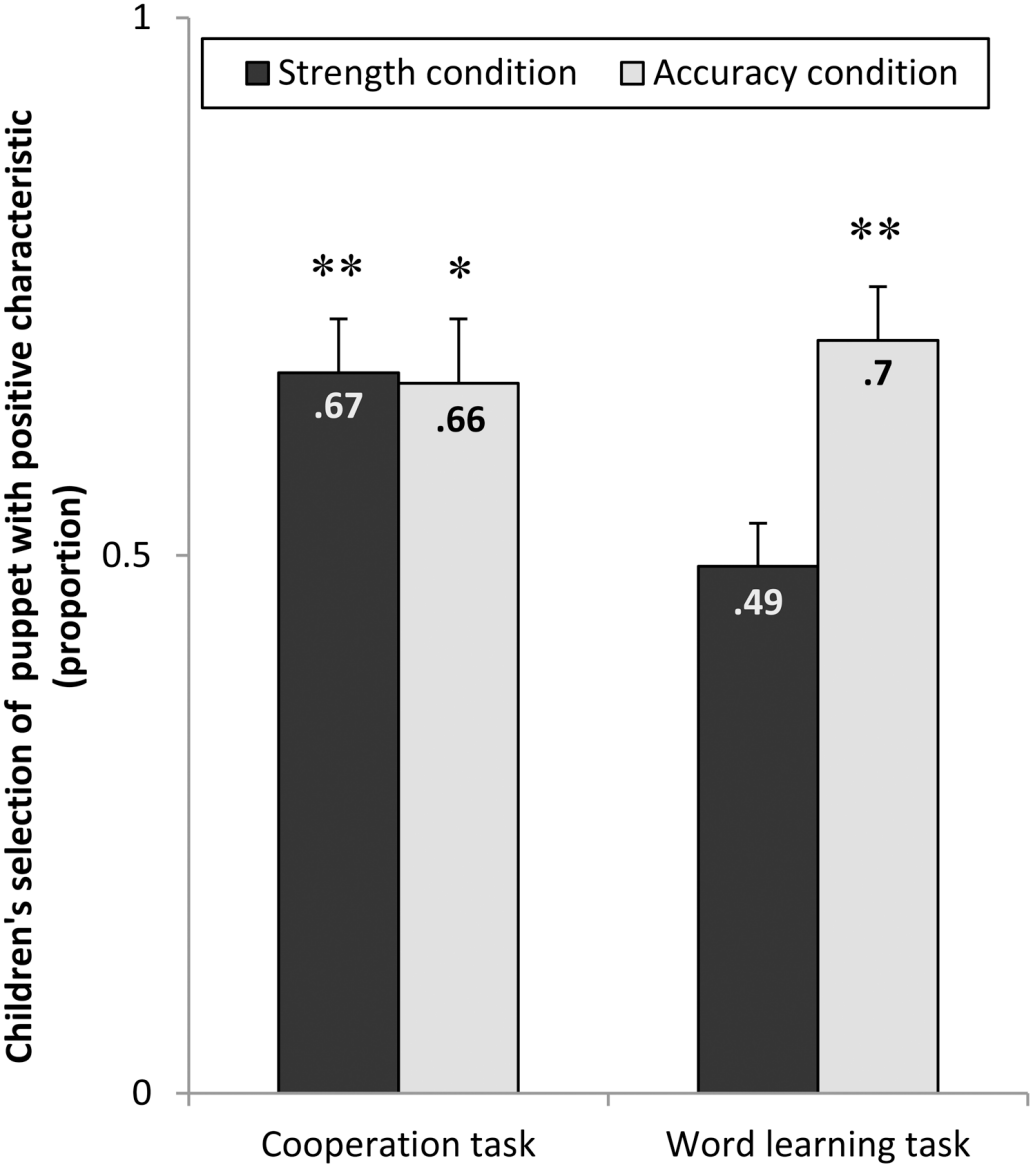
Children’s selection of the more puppet with the positive characteristic. Children’s selection of the puppet with the positive characteristic as a function of task and familiarization condition. Significantly above-chance choice of the model with the positive characteristic (accurate/strong) is marked by asterisks (*p < .05 and **p < .01, one-sample t-tests). Error bars show standard errors.

Children’s choice patterns of the strong/weak or accurate/inaccurate puppets are depicted in Fig 2. A mixed ANOVA with type of task as within-subject factor and familiarization condition and age groups as between-subject factors on the choice of the puppet with the positive characteristic yielded a significant interaction between type of task and familiarization condition (*F*(1,60) = 4.29, *p* < .05, partial η^2^ = .07). No other interactions or main effects emerged. Exploring children’s answer patterns more closely (see Fig 2), the same pattern of rational selective inferences as in adults was revealed: Comparisons of children’s choice of the puppet with the positive characteristic against chance level showed that in the word learning tasks they preferred the accurate puppet’s labels (*t*(31) = 4.12, *p* < .01, *d* = .73), but not the strong puppet’s labels (*t*(31) = 0.2, *p* = .84) above chance, whereas for the cooperation tasks they preferred both the strong puppet (*t*(31) = 3.16, *p* < .01, *d* = .56) and the accurate puppet (*t*(31) = 2.64, *p* < .05, *d* = .47) above chance.

## Discussion

Preschoolers in this study selectively recruited models as cooperative partners and as informants in a systematical and rational way, and in basically the same way as the adults did: they regarded multiple characteristics (strength and knowledge) as diagnostic for a good co-operative partner whereas they regarded only knowledge as diagnostic for a good informant about novel object labels. These findings show that preschoolers’ perception of others does not simply rest upon global evaluation but that children this age infer characteristics in a rational way, and flexibly adjust their criteria for selective recruitment of partners to the requirements of the situation. Children’s pattern of choices suggests that they considered cooperation (in contrast to word learning) as a broader task, requiring more than just the direct physical characteristics (such as strength) needed.

This flexible pattern of model choice, preferring models with certain characteristics for some but not all tasks, is in line with previous research on selective trust where, for example, children reversed their general preference to trust adults over peers when adults had proven unreliable [17] or when children usually know more about the domain in question (i.e. toys, see [26]). This study, together with other findings on selective trust (e.g. [23,24,27]), provides converging evidence for the theoretical claim that children’s model choices are based on rational inferences [28].

In the present study children were confronted with a circumscribed task for the domain of knowledge (word learning task) and with a broader cooperation task for the domain of strength. Since the types of problems were not counterbalanced between domains—no simple, circumscribed stength tasks and no cooperation tasks for which labeling was relevant were administered—the present data cannot strictly rule out an alternative interpretation of the results. In our initial interpretation we distinguished between problems that require differentially broad scopes of characteristics. An alternative—potentially complementary—interpretation of the present results, however, distinguishes between characteristics, rather than problems, of different scope: some characteristics (e.g. knowledge) might be predictive for a wider range of problems (e.g. word learning and cooperation) whereas other characteristics (e.g. physical strength) are predictive for only a limited set of problems directly linked to the characteristic (e.g. physically taxing cooperative activities). This, however, marks a rather rich interpretation in that it presupposes that children understand that characteristics can differ in breadth and hence in inferential scope. Furthermore, recent results suggest that children generally draw similarly wide generalizations from strength and knowledge [23].

How can the present results be reconciled with seemingly diverging evidence, in particular in a quite similar design by Fusaro et al. [22]? In that study children were confronted with a good and a bad lifter (strength condition) or a good and a bad labeler (accuracy condition) and later children chose between those models in a labeling and a strength task and answered several trait and behavioral prediction questions. In Fusaro et al. [22], children generalized strength widely, choosing the strong over the weak model in nearly all tasks, but generalized narrowly from accuracy (only to labeling-relevant behavior and smartness). The different pattern of model choices in the strength-related tasks of the current study was as predicted and reflects the essential difference between these task and the one by Fusaro et al. [22]: Whereas in Fusaro et al. [22] the strength task was exclusively connected to strength (i.e., lifting objects), the cooperation tasks in the present studies were broader in their requirements, extending beyond physical strength. The discrepancy in findings concerning the knowledge-related tasks plausibly came about due to a crucial difference in the familiarization phase between both studies. In the present study, the strong vs. weak models were introduced as a minimal contrast pair such that they only differed in the crucial respect whether or not they were able to lift objects. In Fusaro et al. [22], in contrast, the two models differed not only in their ability to lift, but also in the accuracy of their announcements: both uttered “I will lift this” before successfully/unsuccessfully trying to lift. The weak model was thus not only weak but also inaccurate (in estimating her own capacities). This, in turn, would make broader inferences concerning the contrast between the two models perfectly rational. In our study with the minimal contrast, however, such broad inferences are in no way licensed.

One potential concern regarding the familiarization in the present study is that the manipulations of strength and knowledge were not clearly symmetrical. The inaccurate model’s wrong labels might have been perceived as bizarre and less excusable than the weak model’s failure in e.g. lifting a suitcase, and this might have led to an avoidance of the inaccurate model across the board. However, since in Fusaro et al. [22] the familiarization was similar to the present study and children did not avoid the inaccurate model for strength tasks, it is most likely that children’s preference for the accurate model in cooperation tasks in the present study indicates that they regard a broader range of characteristics as relevant for cooperation as compared to lifting.

One assumption, that both interpretations described above (the assessment of the differential scope of tasks and the assessment of the differential scope of characteristics) make, is that in ascribing characteristics, children engage in two inferences. First, an inference from some observed behavior (e.g. labeling/lifting something) to ascribing traits (”smart”/“strong”) to the agent, and a second inference from the traits to predicting future behavior, such as performance in a given task. In the light of established research on the development of trait understanding, our results (along with recent results on selective trust, see [23]) reveal surprisingly early trait reasoning capacities in children—given that in prior research on trait reasoning children showed rational behavior-to-behavior inferences only at a much later age [29,30] whereas preschoolers showed indications of trait understanding only if the inferential chain was split (e.g. only trait-behavior inferences, see [31]) or if indirect measures, such as their reasoning about mental states, were used [32]. We do not know yet what accounts for the difference between children’s reasoning about abilities in selective trust and about personality traits. One potential difference between these inferences is that personality traits are more abstract than the traits relevant in selective trust research. Children’s reasoning about traits with low degrees of abstraction in selective trust scenarios might present a Zone of Proximal Development for trait understanding, with later development towards successful reasoning about more abstract traits.

## Supporting Information

S1 Dataset SPSS data file of the pilot study (adults). (SAV)

S2 Dataset SPSS data file of the main study (children). (SAV)

S1 Fig Novel objects used in the word learning test block. (PDF)

S1 Table Objects and incorrect object labels used in the accuracy familiarization condition. (PDF)

S2 Table Objects used in the strength familiarization condition and associated actions. (PDF)

S3 Table Apparatuses used in the cooperative test block and associated actions. (PDF)
