# Supplementary figures and images for: Selective Cooperation in Early Childhood – How to Choose Models and Partners
Source: PLoS One. 2016 Aug 9;11(8):e0160881. doi: 10.1371/journal.pone.0160881 (PMC4978381; doi:10.1371/journal.pone.0160881)

**S1 Fig. Novel objects used in the word learning test block.**

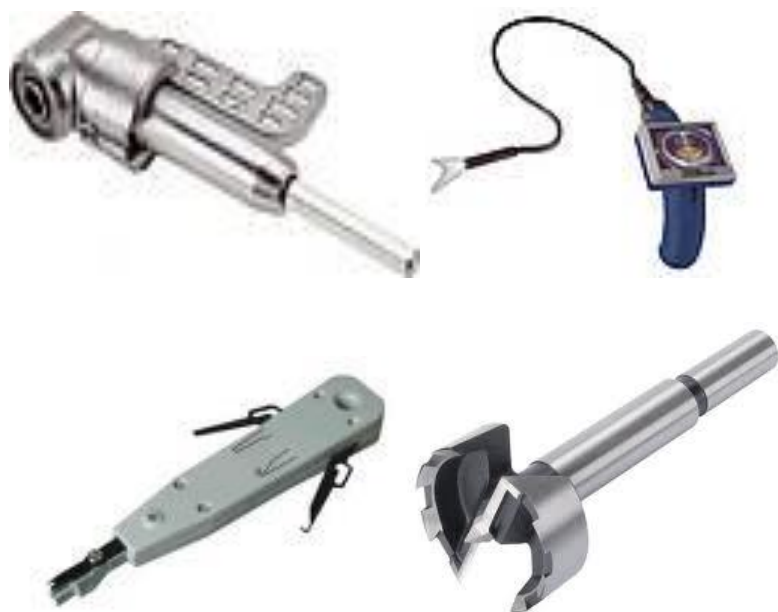

Supplement: S1 Fig — (PDF) [file pone.0160881.s003.pdf]
