# Supplementary material for: Selective Cooperation in Early Childhood – How to Choose Models and Partners
Source: PLoS One. 2016 Aug 9;11(8):e0160881. doi: 10.1371/journal.pone.0160881 (PMC4978381; doi:10.1371/journal.pone.0160881)
Supplement: S1 Table — (PDF) [file pone.0160881.s004.pdf]

S1 Table. Objects and incorrect object labels used in the accuracy familiarization condition.

Object	Label used by inaccurate puppet
Initial familiarization phase:	
Ball	Shoe
Car	Cat
Book	Pants
Cup	Chain
Reminder familiarization phase:	
Flower	Lamp
Chair	Candle
