# Supplementary material for: Selective Cooperation in Early Childhood – How to Choose Models and Partners
Source: PLoS One. 2016 Aug 9;11(8):e0160881. doi: 10.1371/journal.pone.0160881 (PMC4978381; doi:10.1371/journal.pone.0160881)
Supplement: S2 Table — (PDF) [file pone.0160881.s005.pdf]

S2 Table. Objects used in the strength familiarization condition and associated actions.

Initial familiarization phase

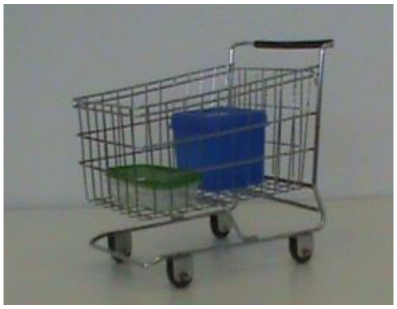

Moving the shopping cart

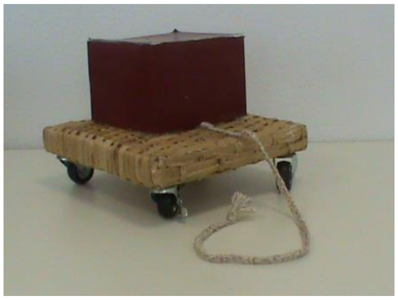

Pulling the box

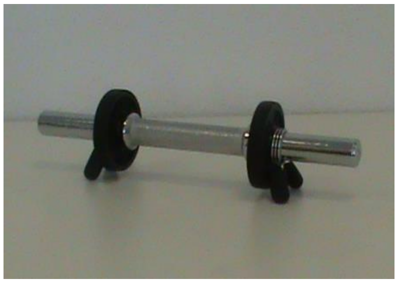

Lifting the dumbbell

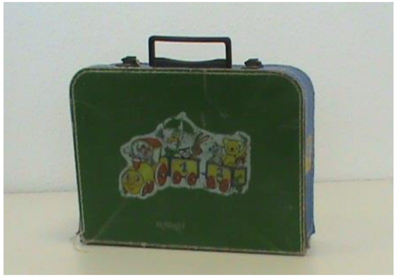

Carrying the suitcase

Reminder familiarization phase

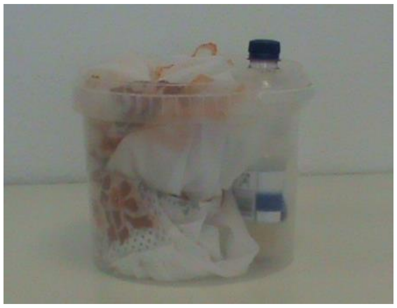

Lifting the bucket

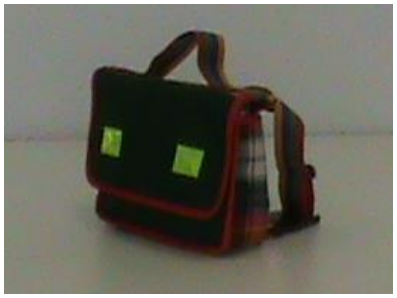

Carrying the backpack
