# Supplementary material for: Selective Cooperation in Early Childhood – How to Choose Models and Partners
Source: PLoS One. 2016 Aug 9;11(8):e0160881. doi: 10.1371/journal.pone.0160881 (PMC4978381; doi:10.1371/journal.pone.0160881)
Supplement: S3 Table — (PDF) [file pone.0160881.s006.pdf]

**S3 Table. Apparatuses used in the cooperative test block and associated actions.**

| Apparatus | Associated action |
|---|---|
| 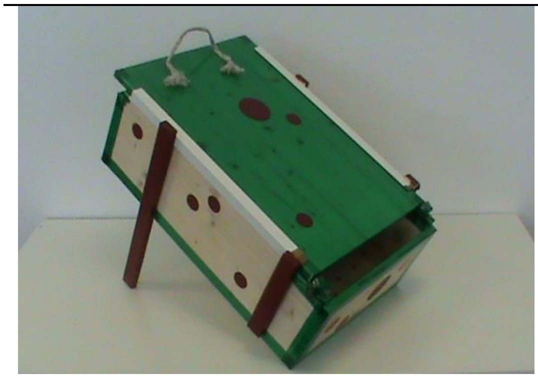 | One actors draws the cord attatched to the green cover upwards, while the other actor extracts the animals hidden inside. |
| 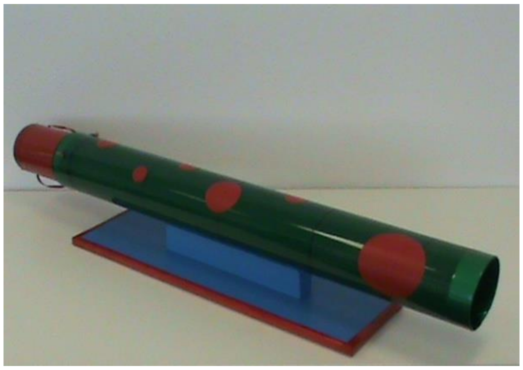 | One actor pushes the red tube inside, while the other actor can retrieve the animals at the green end. |
| 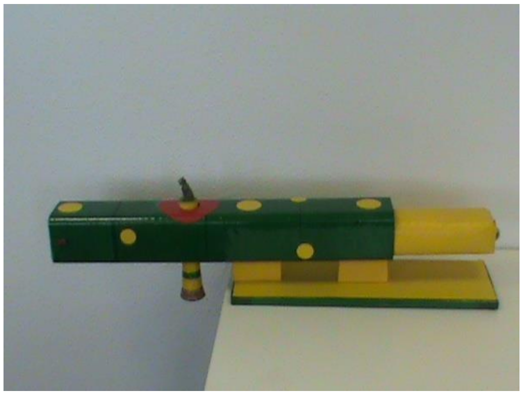 | One actor pulls on the yellow end, while the other actor pushes the green and yellow striped stick upwards and takes the animals that appear on upper side in the red circle. |
| 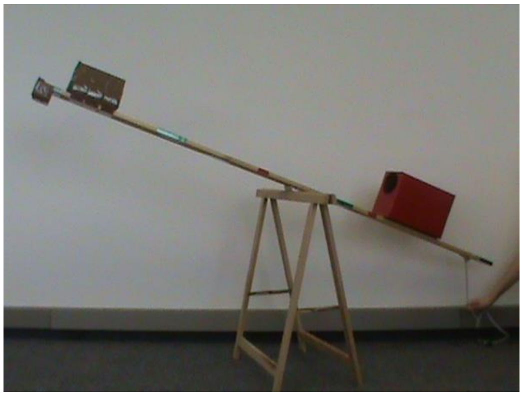 | One actor pulls down the cord on the right end of the rocker while the other actor takes the animals out of the red box. |
